# Digital health systems strengthening in Africa for rapid response to COVID-19

**DOI:** 10.3389/frhs.2022.987828

**Published:** 2022-11-28

**Authors:** Tobias F. Rinke de Wit, Wendy Janssens, Maxwell Antwi, Emmanuel Milimo, Nick Mutegi, Heri Marwa, Njide Ndili, Wasunna Owino, Emma Waiyaiya, Diana C. Garcia Rojas, Monique Dolfing, Aafke de Graaff, Ruan Swanepoel, Mark H. van der Graaf, Dorien Mulder, Teresa De Sanctis, Santa Kratule, Cem Koyuncu, Khama Rogo, Gloria P. Gómez-Pérez, Nicole Spieker

**Affiliations:** ^1^PharmAccess Foundation, Amsterdam, Netherlands; ^2^Amsterdam Institute for Global Health and Development (AIGHD), Amsterdam, Netherlands; ^3^School of Business and Economics, Vrije Universiteit Amsterdam, Amsterdam, Netherlands; ^4^PharmAccess Foundation, Accra, Ghana; ^5^PharmAccess Foundation, Kisumu, Kenya; ^6^Medical Credit Fund, Nairobi, Kenya; ^7^PharmAccess Foundation, Dar es Salaam, Tanzania; ^8^PharmAccess Foundation, Lagos, Nigeria; ^9^PharmAccess Foundation, Nairobi, Kenya; ^10^Medical Credit Fund, Amsterdam, Netherlands; ^11^African Institute for Health Transformation, Luanda, Kenya

**Keywords:** COVID-19, epidemic preparedness, digital, health systems, Africa

## Abstract

The COVID-19 pandemic has painfully exposed the constraints of fragile health systems in low- and middle-income countries, where global containment measures largely set by high-income countries resulted in disproportionate collateral damage. In Africa, a shift is urgently needed from emergency response to structural health systems strengthening efforts, which requires coordinated interventions to increase access, efficiency, quality, transparency, equity, and flexibility of health services. We postulate that rapid digitalization of health interventions is a key way forward to increase resilience of African health systems to epidemic challenges. In this paper we describe how PharmAccess' ongoing digital health system interventions in Africa were rapidly customized to respond to COVID-19. We describe how we developed: a COVID-19 App for healthcare providers used by more than 1,000 healthcare facilities in 15 African countries from May–November 2020; digital loans to support private healthcare providers with USD 20 million disbursed to healthcare facilities impacted by COVID-19 in Kenya; a customized Dutch mobile COVID-19 triage App with 4,500 users in Ghana; digital diaries to track COVID-19 impacts on household expenditures and healthcare utilization; a public-private partnership for real-time assessment of COVID-19 diagnostics in West-Kenya; and an expanded mobile phone-based maternal and child-care bundle to include COVID-19 adapted services. We also discuss the challenges we faced, the lessons learned, the impact of these interventions on the local healthcare system, and the implications of our findings for policy-making. Digital interventions bring efficiency due to their flexibility and timeliness, allowing co-creation, targeting, and rapid policy decisions through bottom-up approaches. COVID-19 digital innovations allowed for cross-pollinating the interests of patients, providers, payers, and policy-makers in challenging times, showing how such approaches can pave the way to universal health coverage and resilient healthcare systems in Africa.

## Introduction

Low- and middle-income countries (LMICs) worldwide were the ones that suffered the largest direct and indirect effects of the COVID-19 pandemic (1–3). This was expected since even under normal circumstances, their fragile healthcare systems often function at the limit of their capacity. At the onset of COVID-19 there was a global covetous stockpile of personal protective equipment's (PPEs), diagnostic tests, and later on of vaccines (4). LMICs were seriously underserved, and the lack of international health solidarity was demonstrated in an even more painful manner than during the previous HIV epidemic (5), leaving the appeals from the World Health Organization (WHO) and others largely unanswered (6). Moreover, the COVID-19 control measures deployed in the Western world were one-on-one transposed to LMICs without adaptations to the local realities, leaving behind and hitting hard the most vulnerable populations (7, 8). For instance, Western settings were dominated by concerns about lack of tertiary healthcare capacity (9), while in most LMICs this type of care is an unaffordable luxury that reaches only a small sector of the population. In fact, in some Sub-Saharan African countries as Nigeria, up to 85% of the healthcare is provided by primary healthcare centers (10) that were largely ignored during the COVID-19 response in Africa. Stringent lock-down measures were replicated in African settings without any economic/social support to help families covering their essential needs (2, 11). In countries as Kenya, over 80% of the labor force is employed in the informal sector (12). The severe mobility restrictions created disproportional economic and social havoc in terms of income loss, food insecurity, mental health, and domestic violence (13–15). Pandemic-related school closures impacted the sexual and reproductive health of many adolescent girls in LMICs and increased the rates of teenage pregnancies and school dropouts (16, 17). As experienced before for HIV, the Official Development Assistance (ODA) did not actively involve private healthcare facilities in LMICs into the COVID-19 response, even though in Africa half of healthcare is delivered through this sector (18–20). Many existing funds for other diseases (HIV/AIDS) were reallocated for COVID-19, instead of new funds replenished (21). In addition, health insurers and other risk-poolers in LMICs never allowed COVID-19 to be part of their coverage (22). Painful examples are abundant of funds provided to African governments that never reached the poor (23).

Today, CDC-Africa reports almost 12 million COVID-19 cases and over 250,000 COVID-deaths (24). According to various models this is likely a 3-fold underestimation (25–27). Reviewed serosurvey data point toward a massive spread of the epidemic in Africa with a 65% infection rate already in the third quarter of 2021. Nevertheless, COVID-19 symptomatology in Africa appears less severe than elsewhere (28–31). The disease goes unnoticed in many settings due to lack of (reported) symptoms. This lack of symptomatology is likely related to a combination of factors, including a relatively young population, and the scarcity of COVID-19 testing, reporting and post-mortem registration (32), but also to a higher activation status of the immune system (33), and potential protective effects from BCG vaccinations (34), malaria infection (35) and cross-protection by non-pathogenic coronaviruses (36). In other words, COVID-19 is circulating in Africa, but its spread is mostly unseen.

Nevertheless, the COVID-19 pandemic has put large pressure on the functioning of African health systems. On the supply-side, healthcare facilities were challenged due to lack of PPEs, limited availability of workforce due to staff illness or reluctance to come to clinics, limited knowledge on guidelines and how to prepare for COVID-19, and supply chain challenges resulting in lack/increasing prices of essential medicines (37). On the demand-side, there was increased client fear of going to healthcare facilities, inability to travel to clinics due to lockdowns and reduced transport availability, and lack of financial resources to pay for care due to job loss and other economic constraints (38). In addition, limited knowledge and awareness due to lack of real-time factual information on the dynamics of COVID-19 significantly undermined health managers' and policymakers' ability to take appropriate policy decisions and undertake targeted action.

All in all, COVID-19 demonstrated all over the world, and particularly in Africa, that improved flexibility and resilience of healthcare systems is direly needed. Conventional interventions aimed at specific diseases (vertical interventions), or at subsets of health systems, have proven to be unsustainable—and sometimes even undermining the general healthcare system (39). In consequence, despite the considerable (inter)national economic investments of the last decades in healthcare in Africa, such efforts have rarely impacted whole healthcare systems, as this rather requires horizontal (sector-wide) interventions. Hence, a paradigm shift is needed: Africa needs better healthcare for money, and more money for better health by embedding disease-specific financing into general healthcare financing systems, such as insurances. A 2019 Brookings report stipulated Africa carrying 23% of the global disease burden yet accounting for only 1% of the total global health expenditures (40). More money is needed in the healthcare system and at the same time more efficiency should be created with the existing money, while shifting from disease exceptionalism to more integrated approaches.

## Assessment of policy options and implications

This article argues that digitalization of African healthcare systems creates numerous mutually reinforcing advantages for all health system stakeholders: patients, providers, policy-makers and payers. It allows for better tracking and tracing of money streams, reduction of corruption, increased transparency, and accountability. In addition, digital healthcare contracts allow for operational efficiency, create trust, and lower transaction costs between patients, payers, and providers. Moreover, digital health systems allow for better and more timely data collection and thus open the potential for more punctual, fact-based policy and decision-making. Digital mobile health interventions enable patient-centered approaches, the democratization of health knowledge, therefore increasing social equity, social inclusion, and patient empowerment. Finally, using digital approaches the current fragmentation and duplication of healthcare financing efforts in LMICs can be counteracted, working toward Universal Health Coverage (UHC) (41). Importantly, the flexibility, scalability, and timeliness of digital interventions make them very suitable for emergency responses to epidemics, transcending geographical and economic barriers.

Currently, demand (patients)- and supply (providers)-side processes in LMICs lead to vicious circles of low coverage, low quality, and low returns in the health system. Financial and technical support for quality improvement (QI) is often lacking. Health facilities perceive investments in QI to be costly rather than as a profitable business priority or social investment. Facilities are thus more likely to provide low-quality services, with consequently low, erratic patient utilization, leading to poor and unreliable revenues and business performance. The latter in turn constitutes an obstacle to obtaining bank loans to improve the infrastructure and services of their facilities. Without the ability to provide good quality services, these facilities have fewer chances to be empaneled by (national) health insurers, and therefore depend largely on out-of-pocket payments that do not offer a predictable and regular source of revenue. Patients, meanwhile, are hesitant to enroll in pre-paid health insurance schemes when quality or benefit packages do not meet their needs or expectations, which makes them vulnerable to catastrophic health expenses and deteriorating health outcomes.

Our organization, PharmAccess Foundation, strives to improve health and access to quality healthcare in Africa with an integrated digital full-system approach to transform the above-described vicious circle into a virtuous circle and create inclusive health markets. Such health systems will be more transparent, real-time, flexible, equitable and efficient, while providing better quality. Our approach is strongly based on mobile technology, as the ubiquity of mobile phones in Africa, even among poor families, provides for the first time in history an opportunity for social and financial inclusion of low-income individuals who are otherwise disconnected from, and unaware of, the central government's decisions, public health information, public services, and international measures. It allows for bidirectional interventions, on the one hand improving the quality of delivered healthcare, while cushioning the economic impact of illnesses and injuries, for instance through digital money or mobile health insurance. Moreover, mobile phones allow for collection of real-time data to inform evidence-based decisions of policy-makers, to promptly respond to population needs, or getting prepared for public health emergencies. All in all, mobile phone-based and real-time data-driven approaches can provide unique additional strength to building digital and resilient healthcare systems and moving toward UHC in Sub-Saharan Africa.

### COVID-19 digital interventions

In this article, we discuss our approaches following the WHO framework for health systems (42), which identifies six building blocks that can be used to evaluate how interventions contribute to health systems strengthening: (1) Health work force, (2) Service delivery, (3) Financing, (4) Medical products, (5) Information, and (6) Governance. The latter two are cross-cutting components that provide the basis for the other four building blocks. For each of the building blocks, we discuss how COVID-19 challenged the health system and describe one digital intervention implemented by PharmAccess that specifically addressed this challenge. Using the results of existing analyses, we found that each of the PharmAccess interventions covered more than one building block; most notably, by their digital nature they all contributed to the strengthening of health information systems and improved decision-making and governance through better and timely data generation. Table 1 summarizes the interventions within the WHO framework and indicates their interconnectedness.

**Table 1:** PharmAccess digital COVID-19 interventions: origin, WHO priority, targeted key stakeholders and health system cross-strengthening potential.

			**WHO priority area of health systems strengthening**						**Contribution to health system circle**			
		**Intervention**	**Health workforce**	**Service delivery**	**Financing**	**Medical products**	**Information**	**Governance**	**Patient**	**Supply**	**Demand**	**Governance and Institutions**
WHO priority area of health systems strengthening	Healthcare workforce	SafeCare4Covid								+		+
	Service delivery	MomCare							+	+	+	
	Financing	MCF Cash Advance								+	+	
	Medical products & technologies	CovidConnect							+	+		+
	Information	Health diaries							+			+
	Governance	COVID-Dx							+	+		+

Light grey boxes represent cross-strengthening; dark grey boxes represent 100% correlation.

#### Health workforce (building block 1)

##### SafeCare4Covid: Improving health provider preparedness for COVID-19

From the onset of COVID-19 in Africa it was perfectly clear that healthcare facilities needed full attention in terms of keeping their staff safe and at work and procuring pertinent supplies and equipment to protect themselves and their patients. Health worker staff, being at the frontline of care, needed protection to avoid facility-based infections and spreading of the disease, as did visiting patients. Providers needed to assess their readiness in terms of staff, supplies (in particular PPEs), equipment, but also to set up and maintain up-to-date processes and knowledge to manage the disease.

To support the knowledge, skills, motivation, and deployment of health staff during the pandemic, we developed the “*SafeCare4Covid* App,” building on the existing digital PharmAccess SafeCare approach (43), an IEEA (ISQua External Evaluation Association)-accredited standards-based, stepwise, quality improvement methodology. *SafeCare4Covid* aimed to prepare and support public and private healthcare workers for COVID-19 worldwide, specifically targeting those in LMICs (44). It helped healthcare personnel to assess their epidemic readiness and provide them with a tailored quality improvement plan (QIP) specifically focused on COVID-19, as well as practical, WHO-based information, posters, and guidelines.

The App is free and was launched in June 2020. Its link (https://covid.safe-care.org) is available worldwide online, and its access is distributed via PharmAccess and its partner organizations to healthcare facilities through email or WhatsApp, or by communicating about the application through webinars or social media. Facility staff could easily create an account to use the mobile app to self-assess, measure and report on the availability of equipment, staff, and basic PPE supplies, as well as check on their own quality processes, protocols, knowledge, and skills to treat patients with COVID-19. The tool could also be used in tablets and computers and gave access to additional resources like “did you know” -questions to combat misinformation and fake news. Facilities were encouraged to implement the changes advised by the tailored QIP they received once their self-assessment was submitted in the SafeCare4Covid App, and to repeat the self-assessment at a later timepoint to track progress. As a result, *SafeCare4Covid* also contributed to improved service delivery (building block 2).

The assessment quantified COVID-19-related capabilities with 31 questions (score range, 0–100), and availability of COVID-19-related essential medical supplies with a 23-supplies checklist (0–100). Facility characteristics and onboarding data were documented as well as important topics like triaging, infections prevention, COVID-19 trainings, staffing, labeling, mental support, and the availability of supplies (PPEs and oxygen). After online submission of the self-assessment, the (meta)data collected was aggregated and shared through comprehensive dashboards with public and private sector stakeholders and donors for informed decision-making on targeting services, human resources, PPEs and additional supplies, equipment to test and treat, inputs for training, quality control and capacity-building (thereby contributing to building blocks 5 and 6). Additional information about the tool can be found in Gómez-Pérez et al.^1^

#### Service delivery (building block 2)

##### MomCare: Supporting client adherence to the continuum of maternal care

Initial COVID-19 predictions painted a bleak picture, particularly for access to care for young mothers and children in LMICs, in line with the experiences from the earlier Ebola-epidemics (45). With schools closing, girls staying home, sexual education programs being suspended, families losing their incomes and increasing domestic violence and rape, the number of teenage pregnancies was surging. Moreover, COVID-19 affected both antenatal care (ANC) and hospital services (e.g., access to ultrasounds, skilled birth attendance, and caesarian deliveries) due to absence, sickness or deployment of staff, curfews, COVID-19 fear and stigma, which endangered mothers' care-seeking behavior and care providers' ability to deliver quality care.

To ensure sustained delivery of maternal and child healthcare services, PharmAccess quickly adapted its so-called MomCare program. MomCare had been implemented since 2017 through the M-TIBA digital health platform (https://mtiba.com/) which links mothers-to-be through their mobile phones with healthcare providers and payers, following a standardized pregnancy journey based on WHO guidelines at a predetermined cost and quality. The program supports low-income women from communities in remote areas and urban settlements in accessing care through a digital wallet on their phone, while facilitating quality upgrades at the health providers and introducing a transparent payment system. The health services along the MomCare pregnancy journey facilitate the detection and reduction of pregnancy-related risk at an early stage to prevent further complications. Based on smart contracting and pay-for-performance financing, MomCare aims to increase transparency in the system, enhance efficiency and increase funding (thereby also contributing to building block 3).

Within the first 3 weeks of the COVID-19 outbreak in Kenya, MomCare added a dedicated set of interventions to the MomCare-bundle to ensure continued accessibility and good-quality care: emergency ambulance services during curfew hours, extended bed allowances to encourage early arrival at the clinic for delivery, phone calls to check on women approaching their delivery dates and discuss birth plans, SMS messages to inform pregnant women of labor and complication signs, of open facilities hours and COVID-19 protocols, and training for clinic staff in managing COVID-19 patients and infection prevention. The digital M-TIBA platform enhanced the ability of providers to reach out to pregnant women at the right time, with the right message, and to build internal capacity for improved safety practices during the pandemic. The newly added interventions aimed to mitigate the effects of the COVID-19 outbreak on women's ability to adhere to the continuum of maternal care and providers' capacity to ensure a safe environment, in a context of greatly reduced mobility due to travel restrictions and fear (46).

Upon enrolment in the MomCare program, and after giving informed consent, women would answer to a baseline survey collecting basic socio-economic information as well as birth history and pregnancy data. At each subsequent visit, women checked in with their phone, and patient data were added in real-time to the data platform (facilitating building blocks 5 and 6). These data enabled us to track the uptake and utilization of the additional services that were offered through the digital platform, as well as key outcomes including the number of ANC visits, the percentage of high-risk pregnancies, of skilled deliveries, and of normal vs. caesarian deliveries.

#### Financing (building block 3)

##### Medical credit fund cash advance: Enhancing healthcare provider access to finance

Quickly after the onset of the COVID-19 pandemic, private healthcare providers in Africa were in urgent need of working capital to ensure the quality and continuity of their healthcare delivery. Patients avoided visiting hospitals and clinics out of fear of being infected or due to transport challenges and curfews, which resulted in a sharp decline in revenues at these facilities. At the same time, the facilities needed funds to purchase PPE and pay salaries to protect and retain their staff and ensure the continuity and quality of care. Healthcare facilities urgently needed working capital to keep themselves afloat at a time when banks were even more wary of providing loans to small businesses. In addition, lockdowns and travel restrictions further complicated healthcare providers' ability to request for financial support.

To address the acute need for capital in times of reduced mobility, we supported providers with digital loans (*Medical Credit Fund [MCF] Cash Advance:* https://www.medicalcreditfund.org/) to invest in their services and/or overcome dips in turnover due to COVID-19.

The MCF had already developed its Cash Advance product in partnership with CarePay in Kenya. Cash Advance is a digital loan based on mobile money revenues (through Kenya's M-PESA) that requires no collateral. Existing MCF customers can apply for a loan via their mobile phones by dialing a specific shortcode that will take them through a USSD (Unstructured Supplementary Service Data) menu. USSD is a global system for mobile communications that allows for two-way messages without requiring to download an app, nor an internet connection. Hence, it is also supported when the customer does not have a smartphone. The client can choose the amount he/she wants to borrow. The loan is disbursed quickly (within 24 h) and automatically repaid from mobile money payments received by the healthcare center. The health facility can decide the percentage of the mobile payment revenues he/she wants to dedicate to pay back the loan: 30, 50, 66, or 100 percent until the loan is fully paid. Cash Advance loans are typically used to address working capital needs or invest in medical equipment.

The MCF was established in 2009 as a blended fund aimed at increasing access to capital for primary healthcare providers in Africa. It was the first debt fund solely dedicated to the health sector in the region. MCF combines loans with technical assistance to allow small- and medium-sized healthcare companies to invest in expansion and quality improvement. So far, MCF has disbursed USD 160 million in loans to over 1,800 healthcare providers across the continent, both through traditional and digital loans. With loan repayment rates consistently above 94%, MCF has proven that the African health sector is bankable.

However, there is still a huge financing gap in the health sector, especially for small businesses, which often do not qualify for bank financing. Addressing this gap, a digital loan product such as Cash Advance was considered valuable during the COVID-19 pandemic. With the country in lock-down, decreased patient numbers and increased costs, many healthcare providers suffered from severe cashflow shortages. Using Cash Advance digital loans, MCF could support these providers and disburse cash quickly, safely, remotely, and efficiently.

#### Medical products (vaccines, and technology) (building block 4)

##### CovidConnect: Early warning and triage of suspect COVID-19 patients

In the early stage of the COVID-19 pandemic, the population had to be informed about symptoms, so they could recognize them and subsequently report for SARS-CoV-2 testing and get access to healthcare. Without people reporting for testing, one remains practically in the dark when assessing geographic hotspots or target populations. For this reason, pertinent action-taking (ringfencing, etc.) would be largely delayed, leading to further exacerbation of the pandemic.

To facilitate self-assessment of COVID-19 symptoms and the need to test, a digital tool, named Luscii (https://luscii.com), was developed and rolled out in the Netherlands in Q2 2020 (47). The tool was quickly adapted, dubbed *CovidConnect App*, and subsequently applied in Ghana (https://www.pharmaccess.org/ghana/ghana-covidconnect-supported-by-luscii-and-pharmaccess-foundation/), Nigeria, and Kenya. The CovidConnect App could be accessed by downloading the Luscii App from the App Store or Google Play, and selecting the country (Ghana, Nigeria, or Kenya). At present it has been taken offline and cannot be downloaded/accessed anymore, since it is no longer needed. The adapted tool empowered users to screen themselves for COVID-19 symptoms and be continuously monitored by trained health professionals through a free call-center linked to a medical professional setting. The goal was to reduce the burden on African health workers and respondents to screen, monitor, and follow up potential COVID-19 cases. CovidConnect functioned as a rapid “triage” to select those with COVID-19 reminiscent symptoms to report at formal healthcare facilities for testing. The program was set-up as a tri-partite partnership between PharmAccess Foundation (planning, design, training, data collection and analyses, including funding for marketing and communication activities); Luscii Technologies, a Dutch company that developed the LusciiApp, and adapted it to the local regulations and standards; and local hospitals that provided clinical expertise, care, logistics and transportation support required in the diagnosis and management of COVID-19 cases.

#### Information (building block 5)

##### Weekly diaries: Tracking indirect economic and health impacts of COVID-19 on low-income households

The impact of epidemics reaches far beyond the direct morbidity and mortality rates caused by the epidemic-disease. From the start of the COVID-19 pandemic, there were major concerns across Africa not only about the continued provision of basic healthcare (antenatal care [ANC], childhood immunizations, malaria treatment) and the health-seeking behavior of low-income households, but also about the economic impacts that the social and mobilization restrictions would have on households in resource-limited settings. Unfortunately, continuous, routine household-level data collection systems are scarce on the continent, impeding the ability of policy-makers to closely track impacts on the ground.

However, as part of an ongoing research study (*Diaries)* assessing the socio-economic effects of our interventions at the African household level, we had a household panel running in rural Kenya which enabled us to immediately and precisely measure the socio-economic and indirect health impacts of COVID-19.

Since the end of 2019, PharmAccess had been collaborating with the African Population and Health Research Center (APHRC), the Amsterdam Institute for Global Health and Development (AIGHD) and Amref Health Africa to collect weekly financial and health diaries data from a representative sample of households with young children in Western Kenya. Data collection consisted of a baseline household survey, followed by weekly interviews using digital data entry tools, with the objective of evaluating the impact of a digital health insurance program (48). Whereas, the roll-out of the program was delayed because of the pandemic, the well-established digital data collection infrastructure allowed us to swiftly move from in-person to phone-based interviewing immediately upon the first case being detected in Kenya. The strong rapport that had been built between enumerators and households ensured a continued high response rate to the phone calls.

All economically active, adult household members—both men and women—were invited to participate in the financial diaries, while the health diaries covered all household members regardless of their age. In total, our *Diaries* data covered 345 households encompassing more than 1,900 individuals, following them from October 2019 until November 2020. The financial diaries recorded all weekly incomes and expenditures, savings, loans, and gifts/remittances given and received; the health diaries recorded weekly health symptoms, consultations, provider type and health expenditures. Thus, over the period of a full year, the financial diaries could track in detail how COVID-19 affected poor households' income-generating capacity, their gifts and remittances, loans, and credit. The health diaries closely monitored any illness symptoms of all household members, whether COVID-related or not, as well as health-seeking behavior and provider consultations. Combined, these two types of high-detail, high-frequency datasets were able to give unprecedented insights into the indirect effects of the pandemic and its containment measures in order to inform and guide policies and social safety net interventions (thereby also contributing to building block 6).

#### Leadership and governance (building block 6)

##### COVID-Dx: Informing health managers and policy makers on COVID-19 epidemic dynamics

Healthcare leadership in Sub-Saharan Africa (SSA) seldomly has a coordinated view on the provision of healthcare through public and private health sector. Moreover, the private sector is rarely involved in (emergency) public health issues. There is a dearth of coordinated public-private healthcare data collection and due to this, policy makers and health managers have limited overview of the actual healthcare capacity in their constituencies. Moreover, data are almost never analyzable in semi-real time, an asset that is dearly needed in times of sudden epidemic outbreaks such as COVID-19.

To immediately scale the health sector potential to respond to an as yet unknown pandemic, PharmAccess supported the Department of Health in Kisumu by adding 9 private healthcare facilities to the ongoing network of COVID-19 response sites. Trainings were provided to the private facilities to comply with COVID-19 Guidelines, patient sample collection and transport to central laboratories of KEMRI organized, SARS-CoV-2 testing was supported and results were rapidly back-reported to patients and health authorities for subsequent contact tracing and quarantine measures. This effort was supported by a fully digitalized patient journey tool, COVID-Dx. The aggregate data of COVID-Dx were rapidly translated into digital dashboards (building block 5) that became password-protected available on the mobile phones, tablets and computers of healthcare workers and pertinent stakeholders, to facilitate making informed decisions while coping with the pandemic. Subsequently, during the SARS-CoV-2 Delta variant outbreak, and on demand of the Department of Health, COVID-Dx expanded to 33 public and private facilities in Kisumu County. Finally, this effort was further scaled to 84 facilities covering 14 Counties in the so-called LREB (Lake Region Economic Bloc). The COVID-Dx intervention is described in detail elsewhere (49–52).

COVID-Dx importantly contributed to keeping up the capacity of healthcare staff (building block 1). The project specifically targeted healthcare staff with regular COVID-19 testing, to keep up morale, reduce fears and isolate staff as soon as required. COVID-Dx markedly improved the outcomes and experience of COVID-19 service delivery (building block 2), particularly after rapid diagnostic testing became possible. The extensive COVID-19 health information system (building block 5) guided supply management of diagnostics tests, vaccines, ambulances, oxygen, and hospital beds (building block 4).

Many of the above interventions were supported by the mobile healthcare exchange platform M-TIBA, which is providing real-time insights in healthcare transactions and has been described elsewhere (41).

#### Data protection

PharmAccess Foundation strives for compliance with applicable privacy laws and regulations, including GDPR (General Data Protection Regulation) and local African laws, and upholds a high duty of care when it comes to the privacy of all persons involved in PharmAccess programs, and the processing and protection of their personal data. Several technical and organizational measures secure its information and communication technologies (ICT) environment. These include setting up firewalls and intrusion protection systems, encryption, ongoing monitoring, periodical updates and patches, strict user management procedures, multi-layered verification, and a strict password policy. Personal data is only collected and processed when necessary. If possible, data is pseudonymized, and data is removed or anonymized once the processing purpose is completed. Data is stored either encrypted or anonymized. In the case of longitudinal studies such as the Diaries study, where households are followed over an extended period of time, personal identification data are kept securely by the field work team until finalization of the study, after which the data are anonymized. See Abajobir et al. (48) for more details. In case personal data is transferred from Africa to the Netherlands for further analyses, this is done on a pseudonymized or anonymized basis. The cloud services used are Microsoft Azure located in the EU and include built-in security services such as automatic encryption, automated smart traffic monitoring and profiling and smart access control.

For processing activities of data relating to health care programs and activities in Africa, PharmAccess relies on the consent of the data subjects as the lawful basis and sees to it that such informed consent is obtained through the use of extensive consent forms clearly explaining the details of the specific program and which specific personal data is collected and how it is used. In case third parties are needed for the processing of data, data processing agreements are put in place to govern such processing and to ensure that the personal data concerned is protected and all GDPR and local legal requirements are met.

### Assessment of the interventions

Following the six WHO building blocks, this section will describe for each of the selected case interventions: (i) how it addressed the challenges caused to the health system by COVID-19, including uptake, strengths and weaknesses, implementation challenges, and lessons learned; (ii) which insights emerged from the digital data; and (iii) implication of our findings and experiences for evidence-based policy-making.

#### Health work force: *SafeCare4COVID*

The *SafeCare4COVID app* was available quickly after the start of the pandemic, since an already existing self-assessment tool could be adapted. This proved to be a big advantage for healthcare facilities in LMICs, often located in remote areas. It brought reliable, practical, and real-time information based on WHO standards and guidelines, and the means to do a gap-analysis on their clinical, management and quality of care processes at the outset of the pandemic, a period with many uncertainties. In addition, its pre-existence ensured local ownership, and the flexibility of its digital nature facilitated providing to the needs of different partners during the course of the pandemic. Importantly, facilities also responded in an open and truthful manner to their self-assessment, hence providing valuable data on a facility- and aggregate-level on dashboards. The latter allowed informed decision-making and could directly trigger interventions at the network-level, like targeted training, resources, and supplies. This proved to be beneficial in many ways. For instance, it facilitated uptake amongst healthcare providers that were recommended to use it through their networks, ensuring large coverage. Hence, usage was highest in these groups of network-based facilities.

During the later phases of the pandemic, renewed use of the *SafeCare4COVID App* remained relatively limited. Whilst there was still a great need to focus on the improvement of the identified gaps, facilities needed to be stimulated by their networks to use the tool. Follow-up assessments were performed only when requested. Documented boundaries were staff turnover and the challenge to prioritize the self-assessment over the struggle to keep the facility in business during the pandemic.

**Figure 1:**
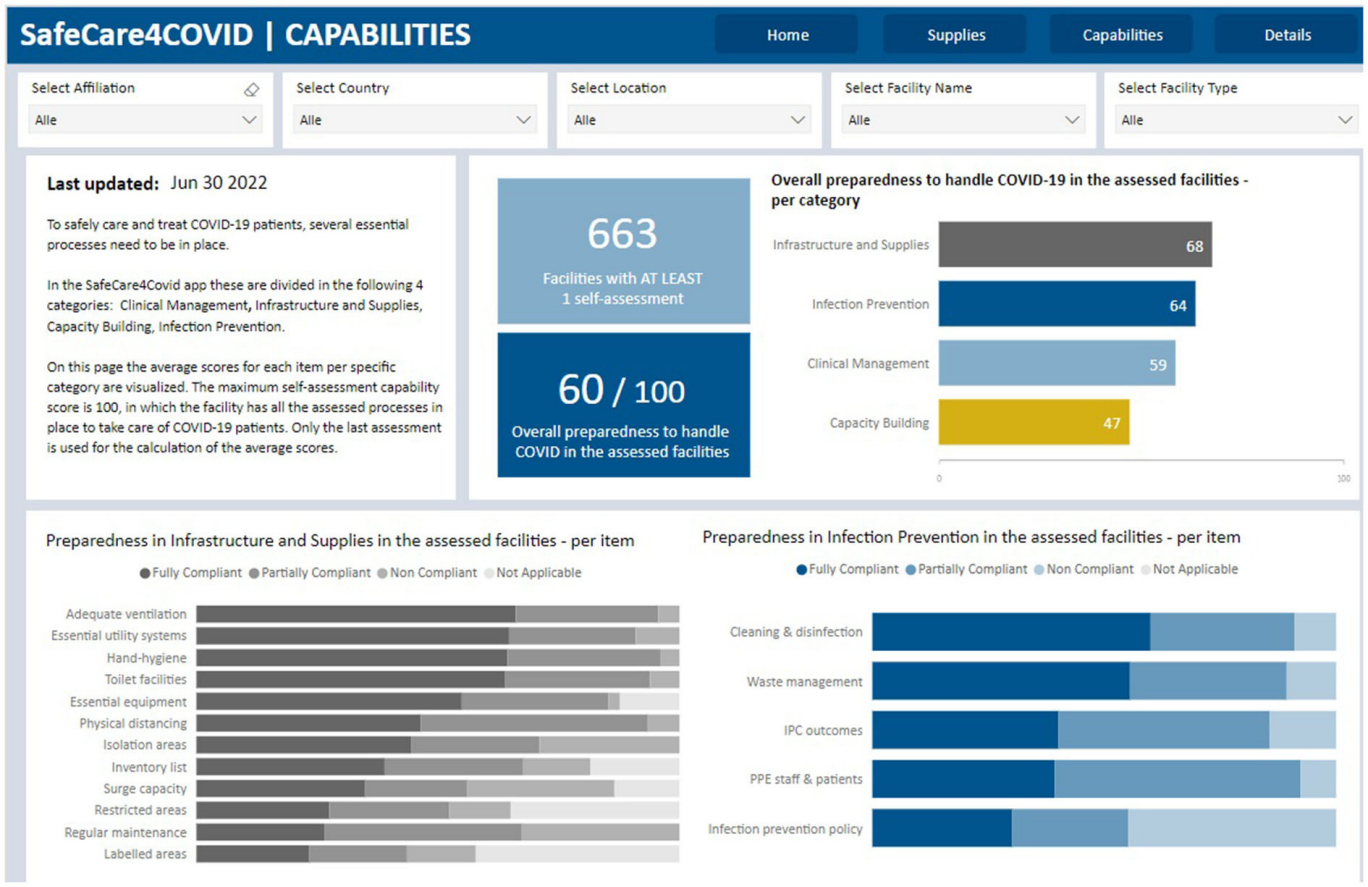
Screenshot of dashboard SafeCare4Covid app showing Capabilities score of healthcare facilities.

**Figure 2:**
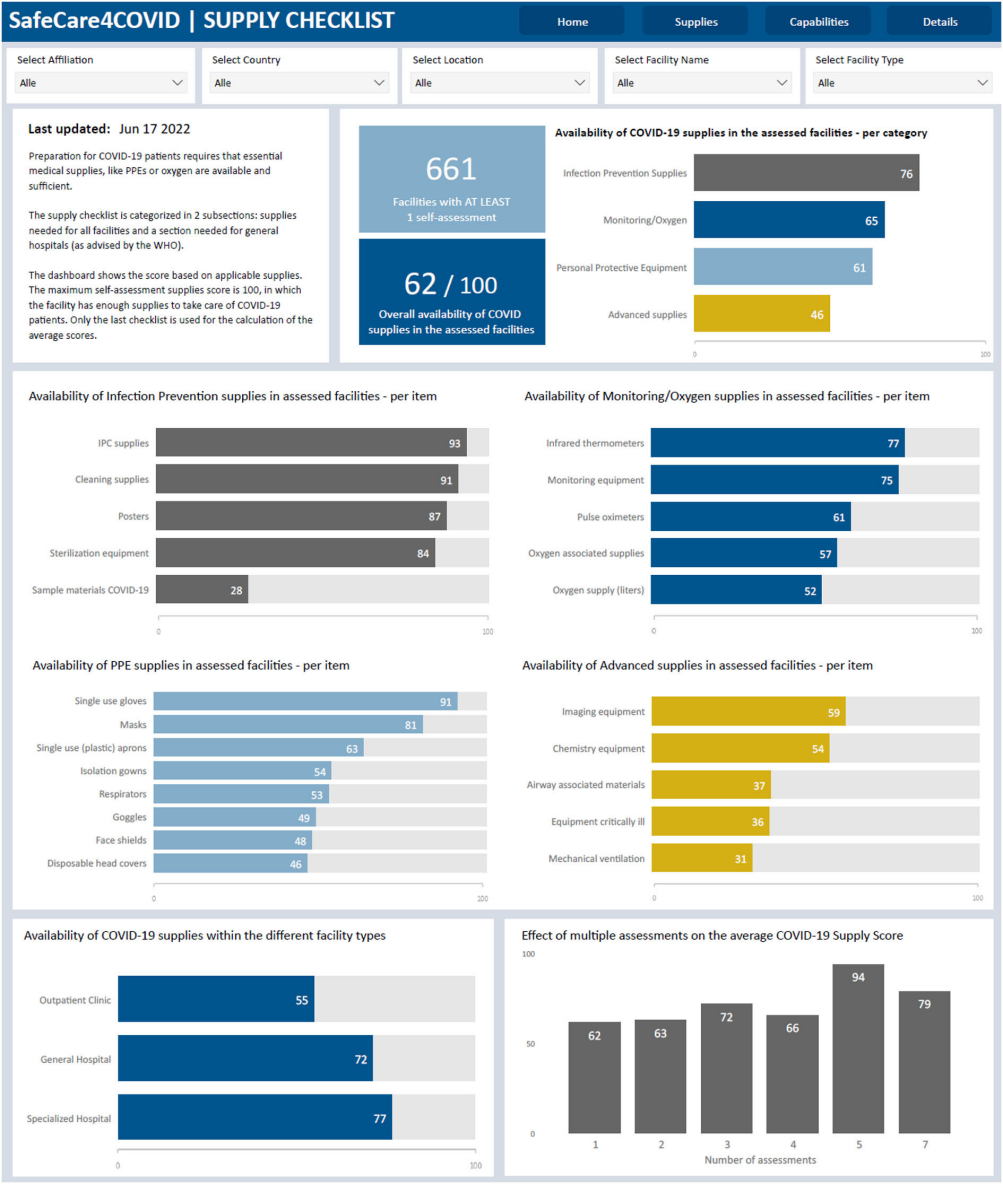
Screenshot of SafeCare4Covid dashboard showing the aggregate information from the supply checklist.

Up to date, more than 1,000 facilities registered on the SafeCare4Covid tool in 19 countries, of which 15 countries are in Africa. A total of 909 self-assessments were performed, with 654 facilities performing a single assessment and 99 facilities performing multiple assessments, as well as the monitoring of capabilities (Figure 1) and essential medical supplies (Figure 2) in these facilities. Eleven partner organizations used the App in their network (amongst which Aga Khan-Kenya, Marie Stopes-Ghana, and CSSC-Tanzania). Serious challenges were found in infection prevention and control, capacity building and clinical management. Timely and practical support, transparency and access to real-time quality information have been shown to be imperative for evidence-based decision-making and will be crucial for improving quality of care and epidemic preparedness beyond COVID-19.

#### Service delivery: *MomCare*

*MomCare* was quickly adapted for COVID-19, providing emergency ambulances during curfew, extending bed allowance for skilled delivery, alerting care providers to high-risk mothers-to-be, sending SMS messages to educate expectant mothers and training care workers to address the pandemic. The digital platform was essential in this respect, providing active phone numbers for direct patient-provider contact.

**Figure 3:**
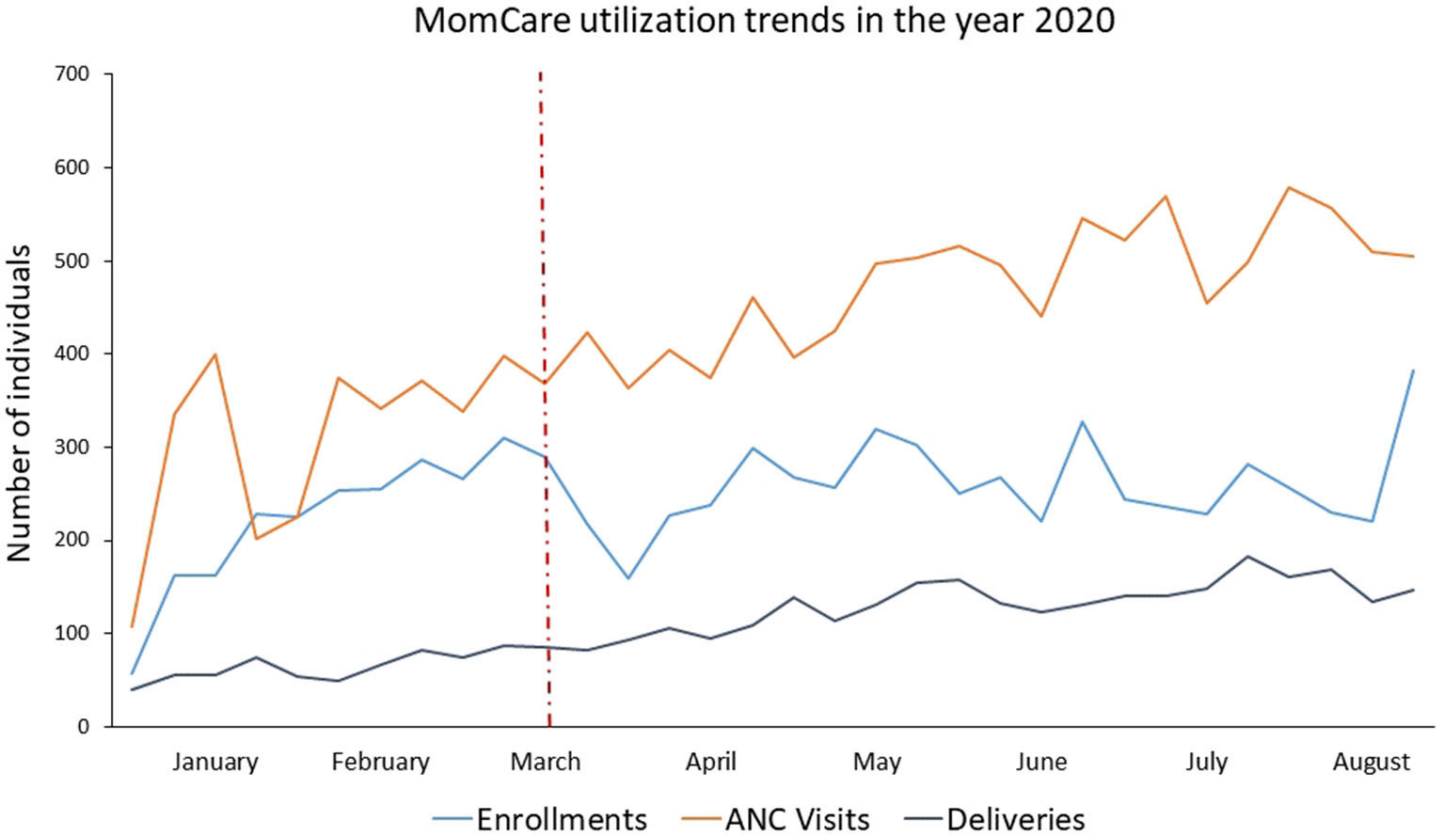
Maternal care utilization dynamics over time before and after COVID-19. The months illustrated in the figure refer to the year 2020. The drop of utilization observed in this graph is due to the reach of the target for the enrolling cohort. ANC and delivery trends did not drop due to the MomCare COVID-19 support interventions, already active 1 week after the first case of COVID-19 was reported in Kenya on 13 March 2020 showed by the dotted, red, vertical line. ANC, antenatal care.

To assess the effect that COVID-19 had on the care journey, we looked at cross-sectional data from 13,443 expectant mothers enrolled in MomCare. We compared data collected through the MomCare platform during the 6 months prior to the first confirmed COVID-19 case in Kenya (September 2019 to February 2020) with data collected during the 6 months that followed; across 26 MomCare clinics concentrated in 3 Kenyan counties: Nairobi (10), Kisumu (12), and Kakamega (3). The results show that care-seeking behaviors (enrollment, antenatal/postnatal care, skilled deliveries) continued to increase for mothers-to-be enrolled in MomCare during the COVID-19 lockdowns, while quality of care and outcomes were maintained (Figure 3). In other words, adherence to the continuum of maternal care was not disrupted among MomCare clients. Public health practitioners could promote interactive, patient-driven technology like MomCare to augment traditional responses, quickly linking donors with patients and providers in times of crisis. For detailed findings, see De Sanctis et al. (53) and Aksünger et al. (54).

#### Finance: MCF digital cash advances

**Figure 4:**
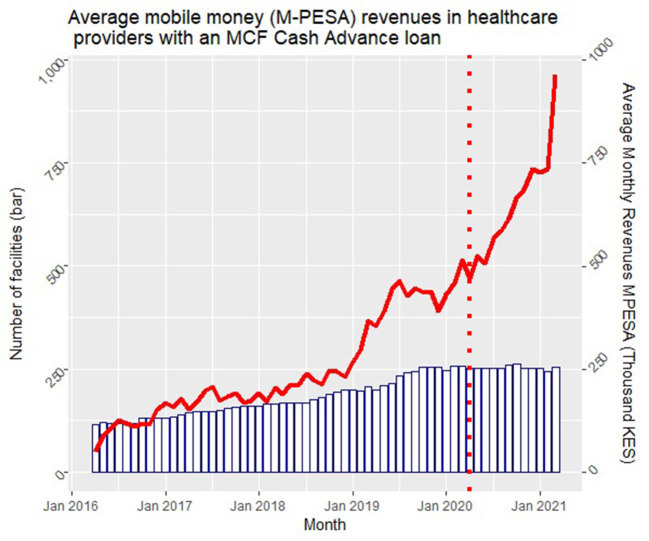
MCF Cash advance. In this figure the bars represent the number of clients with an MCF Cash Advance loan in each given month, while the red line indicates the average monthly M-Pesa payments in these clinics. MCF, Medical Credit Fund.

The MCF digital Cash Advance loans proved highly instrumental for private healthcare providers to bridge gaps in their liquidity during the pandemic: The loans were based on mobile money (M-Pesa) revenues, could be accessed from a mobile phone, did not require collateral, and could be disbursed quickly. In Kenya, the MCF provided 1,109 loans totalling USD 20 million in 2020 through its digital Cash Advance product, reaching 250 healthcare providers throughout the country. Excluding pharmaceutical companies (some of which are very large with equally large loan amounts), cumulative disbursements to dispensaries, health centers and hospitals reached 585,000 Kenyan shillings (KES) (almost USD [US dollar] 5 million) by the end of 2020, and 2,145,000 KES (USD 18 million) 1 year later, in December 2021 (Figure 4).

**Figure 5:**
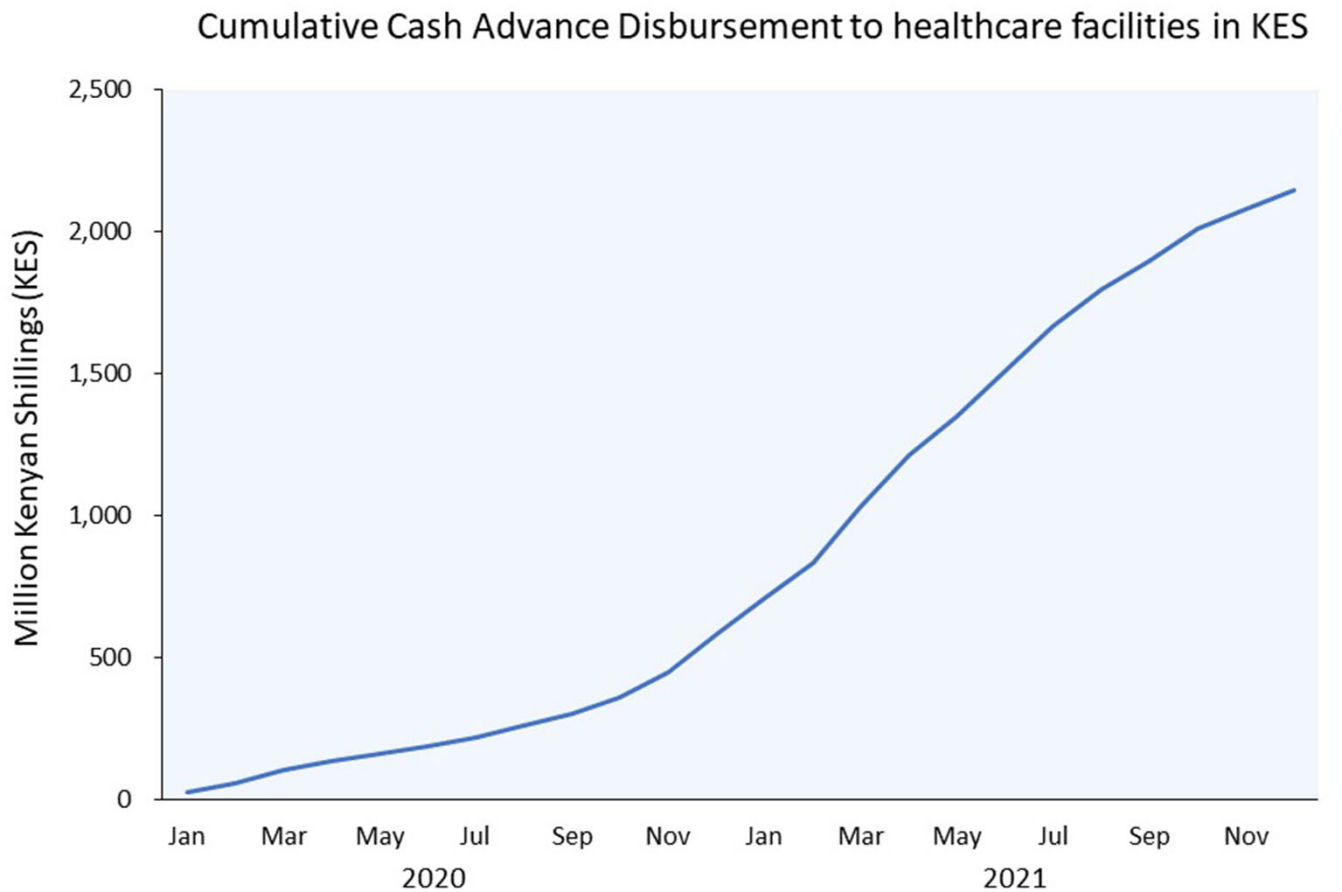
MCF increase in disbursement value in 2020. KES, Kenyan Shilling; USD, US dollar. 1 USD = 116.8 KES (July 4, 2022).

The increase in disbursements was to a large extent enabled by a steady increase of mobile money as a means of payment at healthcare providers, as illustrated in Figure 5. Despite a small dip in the first weeks of the pandemic, digital revenues recovered very fast and kept on increasing during 2020. The use of digital money was actively promoted by African governments during the pandemic; in Kenya, transaction fees for mobile money payments were decreased and fees for mobile money to bank payments and vice versa were waived, while the use of notes and coins was strongly discouraged, as they were identified as potential transmitters of the virus. In addition, Safaricom increased the daily mobile money transfer limit, which further facilitated digital payments at healthcare providers (55).

These increased digital revenues in turn enabled healthcare providers to get larger Cash Advance loans as their credit limits increased concomitantly. While many commercial lenders were shying away from the perceived risk of lending to health facilities, MCF effectively filled this gap and increased its disbursement to private healthcare providers that were not accessing highly needed capital from other lenders. As such, it helped to keep the private healthcare infrastructure afloat during COVID-19 by helping providers to bridge cash flow gaps related to infection peaks and lockdowns. Meanwhile, repayment rates remained consistently high at 94% or more. During the height of the pandemic, repayment of loans was slightly delayed, although this recovered when patients started visiting facilities again.

#### Access to essential medicines and technology: *CovidConnect*

This service was officially launched in Ghana on April 23, 2020, in partnership with the University of Ghana Medical Center and the Ministry of Health. The service was launched in Kenya on July 2, 2020, at Maseno University City Campus grounds, with support from County Government of Kisumu. The service was launched in Nigeria on the June 3, 2020, at Federal Medical Centre Ebute-Metta in Lagos, Nigeria. Outreach to communities was organized by running publicity campaigns, combining informing hospital staff and patient population, with mass media approaches (television interviews, launch events with government officials) and social media campaigns (endorsement by influencers and high-profile celebrities) to support the smartphone solution.

**Figure 6:**
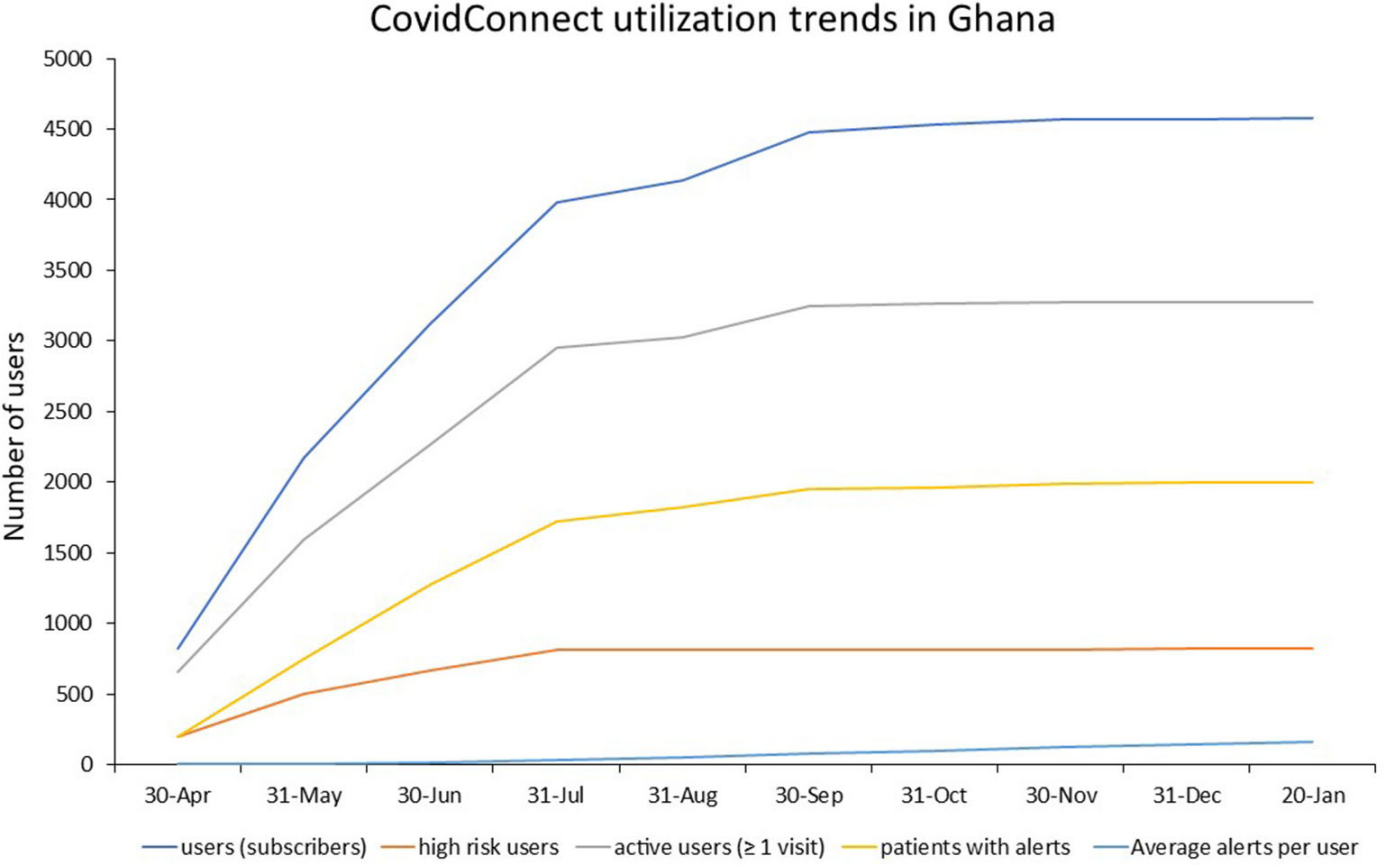
Utilization of CovidConnect in Ghana from April 2020 to January 2021.

By January 2021 in Ghana 4,577 users subscribed to the service and there were 3,274 active users on the platform; 819 were high risk users, 746,760 patient alerts had been processed by the Care Coordination Centre (Figure 6). The marketing and communications campaign in Nigeria recorded an engagement of over half a million persons across all digital platforms. This resulted in a reach of 230,714 and 42,962 application downloads within 6 months. The campaign grew the registered user base month-on-month by 120%, and the weekly active users by 112%. Despite 5,000 daily active users on the platform however, only 23 users tested for COVID-19 and only 8 were diagnosed COVID-positive. In Kenya by mid-October 2020 only 800 users had subscribed to the service, 650 active users on the platform, 195 were high risk users and 2,018 patient alerts were processed by Care Coordination Centre.

Although the *CovidConnect* tool demonstrated initial incremental usage, particularly during early roll out in Ghana and Nigeria, after several months the service appeared less frequently utilized and plateauing was observed. This coincided with a decrease of the COVID-19 burden in the country and therefore presumed reduced appetite for its use. Also, having COVID-19 appeared increasingly related to stigma and therefore less willingness to use the App. Moreover, the App was only accessible on smartphones, which are owned by a minority of more affluent Ghanaians. Since COVID-19 first manifested itself in the richer segments of the population, the App served its purpose. Later, when COVID-19 became more generalized, its use case declined.

In response, it was decided to discontinue the service in all three countries. In Kenya, it officially ended in September 2020; in Ghana, the service was discontinued in October 2020; and in Nigeria in December 2020.

Recently it was decided to repurpose the CovidConnect App to its original features (a non-communicable diseases [NCD] monitor) and customize it for application in Ghana.

#### Health information system: *Diaries*

The ongoing weekly digital *Diaries* data collection provided for an existing panel of households, eliminated the need to request extensive ethical clearance, develop a new sampling frame and collect household contact details, which would otherwise have been required in order to gain household-level insights. Moreover, the built-up rapport with participants allowed us to quickly move to phone-based interviewing without substantial disruptions in data collection when lockdowns and travel restrictions were installed. However, women with their own sim-cards can easily borrow a phone from husband or neighbor in normal times, they needed their own mobile device for the interviews during lockdowns and travel restrictions. Once we realized this, we distributed (low-cost) feature phones to women who did not possess one themselves to secure continued interviewing.

**Figure 7:**
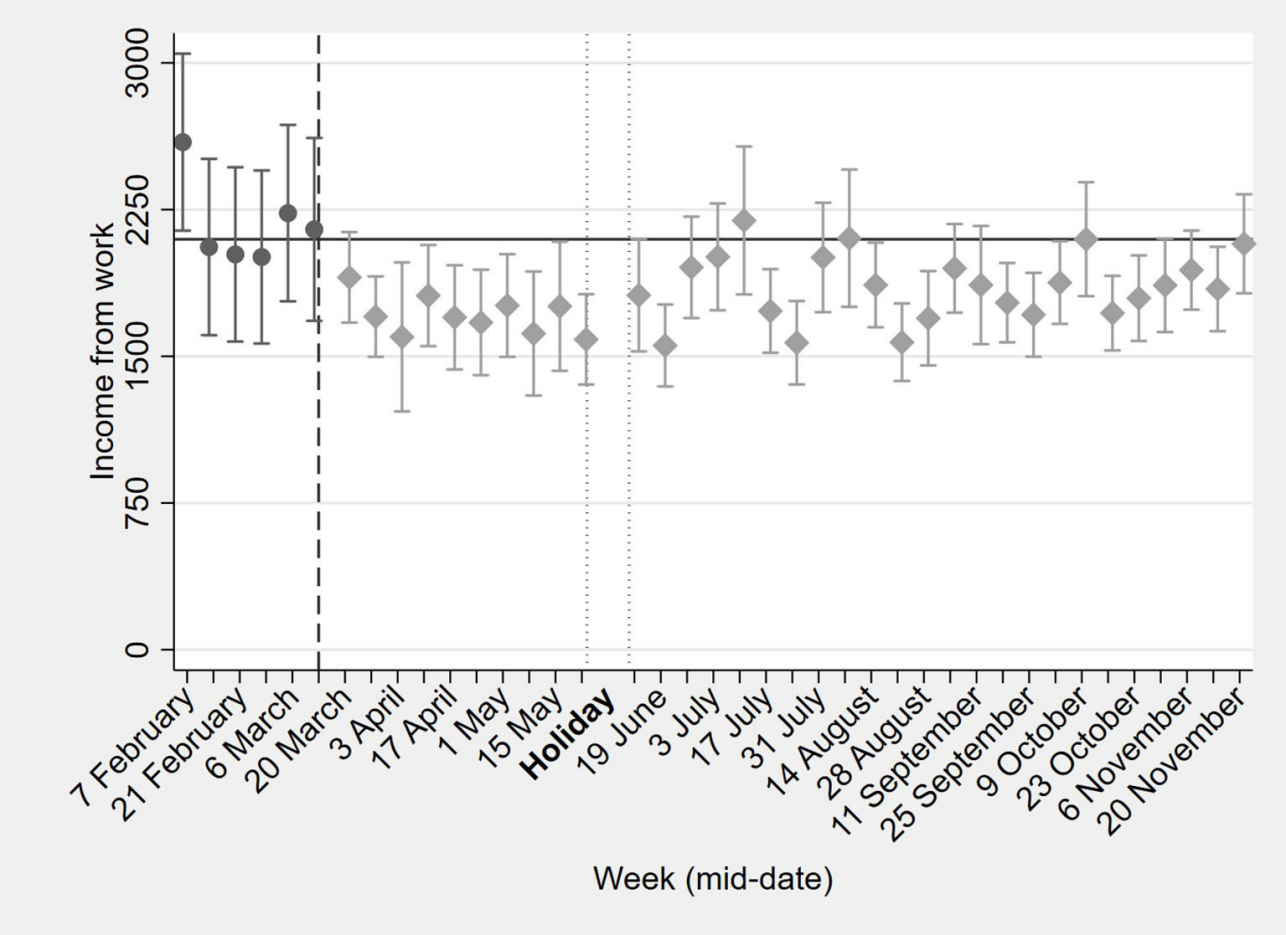
Effects of COVID-19 on household income from work in 2020. “Holiday,” represent the period during which the interviews were suspended due to the holiday season.

**Figure 8:**
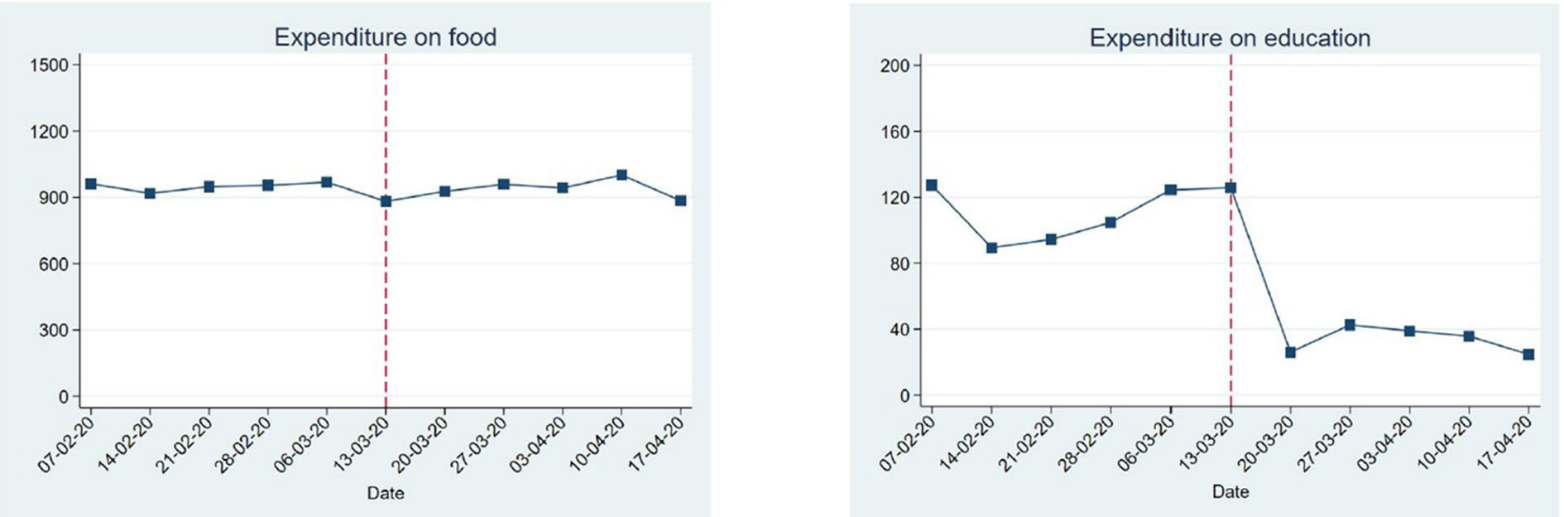
Short-term effects of COVID-19 on household expenditures on food and education. This figure is based on a preliminary analysis performed early during the pandemic, year 2020.

The data gave granular insights into the dynamic impacts of COVID-19 over time for low-income households, which are difficult to distill from larger-scale and less frequent surveys. Many have questioned the tough enforcement of the restrictions. Indeed, our *Financial Diaries* data suggests that overly curbing mobility leads to dramatic drops in income-generation. The financial and health diaries revealed that in the short-term (the first 5–6 weeks after the start of the pandemic), the income from work in the study region decreased with almost one-third and income from gifts and remittances reduced by more than one-third (Figure 7) (56). Households initially managed to keep their expenditures on food at pre-COVID levels. Instead, they spent less on education, gifts and remittances, lent less money to others and postponed loan repayments, suggesting that social support systems (temporarily) broke down (Figure 8).

In the long-term (over the period of a full year), changes were more dynamic, but financial effects of the pandemic lingered on. After the initial drop in incomes, a recovery followed which lasted until mid-August 2020. After that, incomes fell again and remained below pre-COVID levels until the end of the year (Figure 7). These drops in incomes were mostly likely due to the installed curfews. To cope with the income loss, households shifted their employment from salaried work and informal business toward casual labor from mid-August onwards. Nevertheless, our findings indicate that after 8 months, incomes and expenditures in rural communities in the west of the country were close to pre-COVID-19 levels. This suggests a resilience within rural Kenyan communities to deal with the pandemic. Meanwhile, expenditures on education dropped substantially below the pre-COVID-19 levels of KES 176, in line with extended school closures, and never really recovered until the end of November.

Since the first COVID-19 case was reported in Kenya in March 2020, and we started collecting data in October 2019, we were able to study the impact of the COVID-19 pandemic and the containment measures, that included school closures and working from home. We measured the incidence of self-reported medical symptoms and healthcare seeking behavior, including foregone care and shifts to informal care. Moreover, we were interested in knowing if the information we collected differed or not from the official records of district health information services in Kenya (DHIS2).

**Figure 9:**
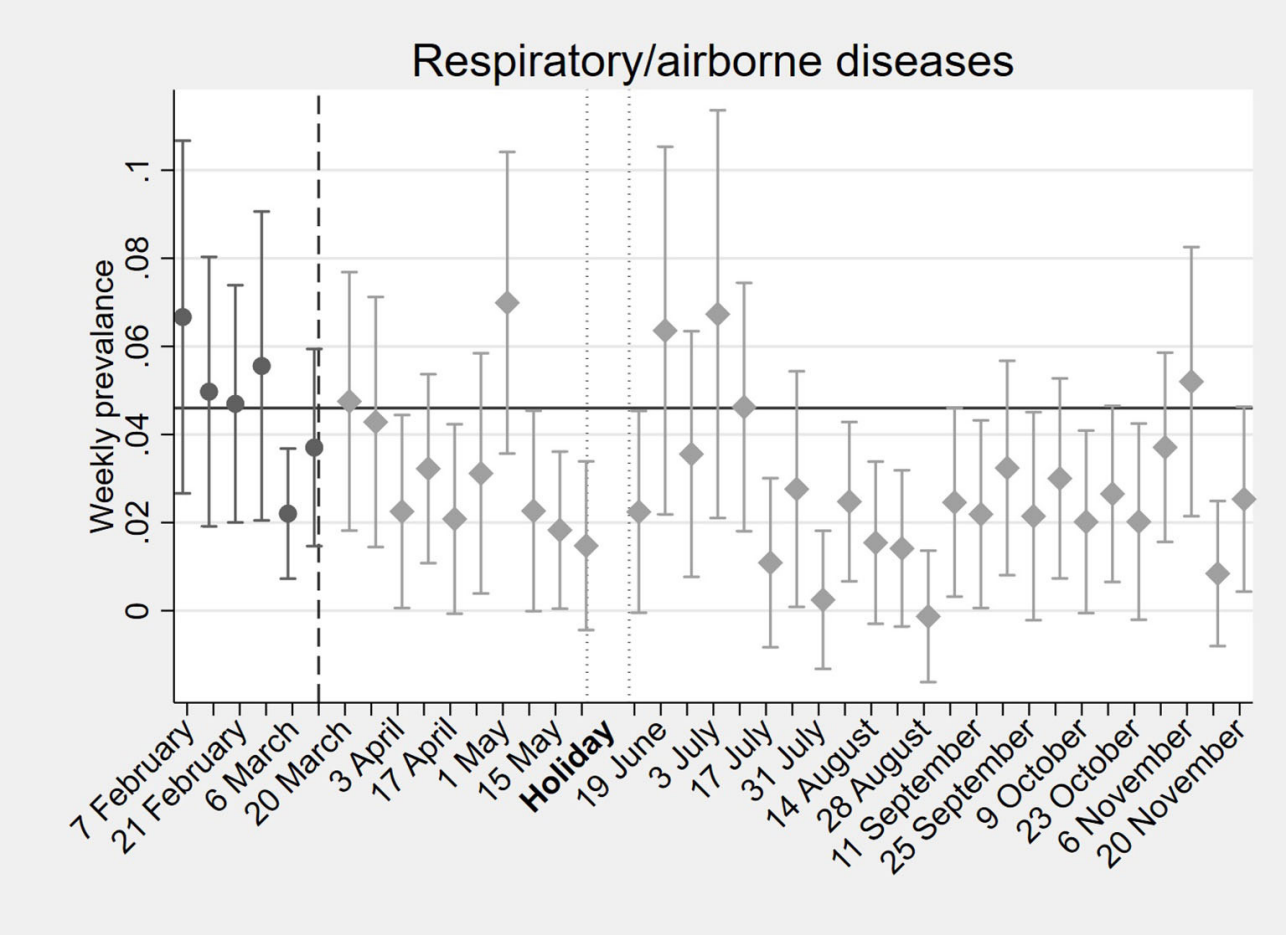
Weekly prevalence of respiratory/airborne diseases in Kisumu, Kenya, before and after the onset of the pandemic in 2020.

According to both our diaries and DHIS2 data, after the onset of the COVID-19, there was a decrease in respiratory infections (Figure 9), enteric illnesses, and malaria or fever. This reduction was observed mainly among children. For adults, incidence of communicable diseases/symptoms rebounded after COVID-19 restrictions were lifted, while for children the effects persisted. Interestingly, both our diaries and the Kenyan DHIS2 data registered a sustained decline in malaria infections, despite the prediction that malaria cases would increase to alarming numbers during the pandemic due to the potential interruption of malaria control measures. Other malaria-endemic African countries report similar findings (57–60).

We found that the reduction in respiratory and enteric illnesses was due to a lower incidence of these infections rather than decreased utilization of healthcare services. This could be explained by reduced spread of such infections linked to limited social contact due to curfews, while schools remained closed for the rest of the year. Finally, in contrast to expectations, the COVID-19 pandemic did not lead to significant increases in foregone care. However, a small shift was observed from care at healthcare facilities to for instance medicine vendors/pharmacies.

The full economic and health impacts of the novel coronavirus pandemic might not be evident in this study period alone but may manifest themselves also through the loss of productive assets or bankruptcy of informal businesses, as well as long-term morbidity due to reduced access to health care for non-COVID- 19 conditions, long COVID-19 or mental health issues caused by the pandemic. These direct and indirect health effects could have knock-on effects on adults' productivity, hence, their ability to contribute to the economy much further down the line. Finally, the school closures have undermined children's educational attainment potential, reinforcing the intergenerational cycle of poverty. The data collection tools were not designed and prolonged to capture such longer-term effects.

#### COVID-Dx

**Figure 10:**
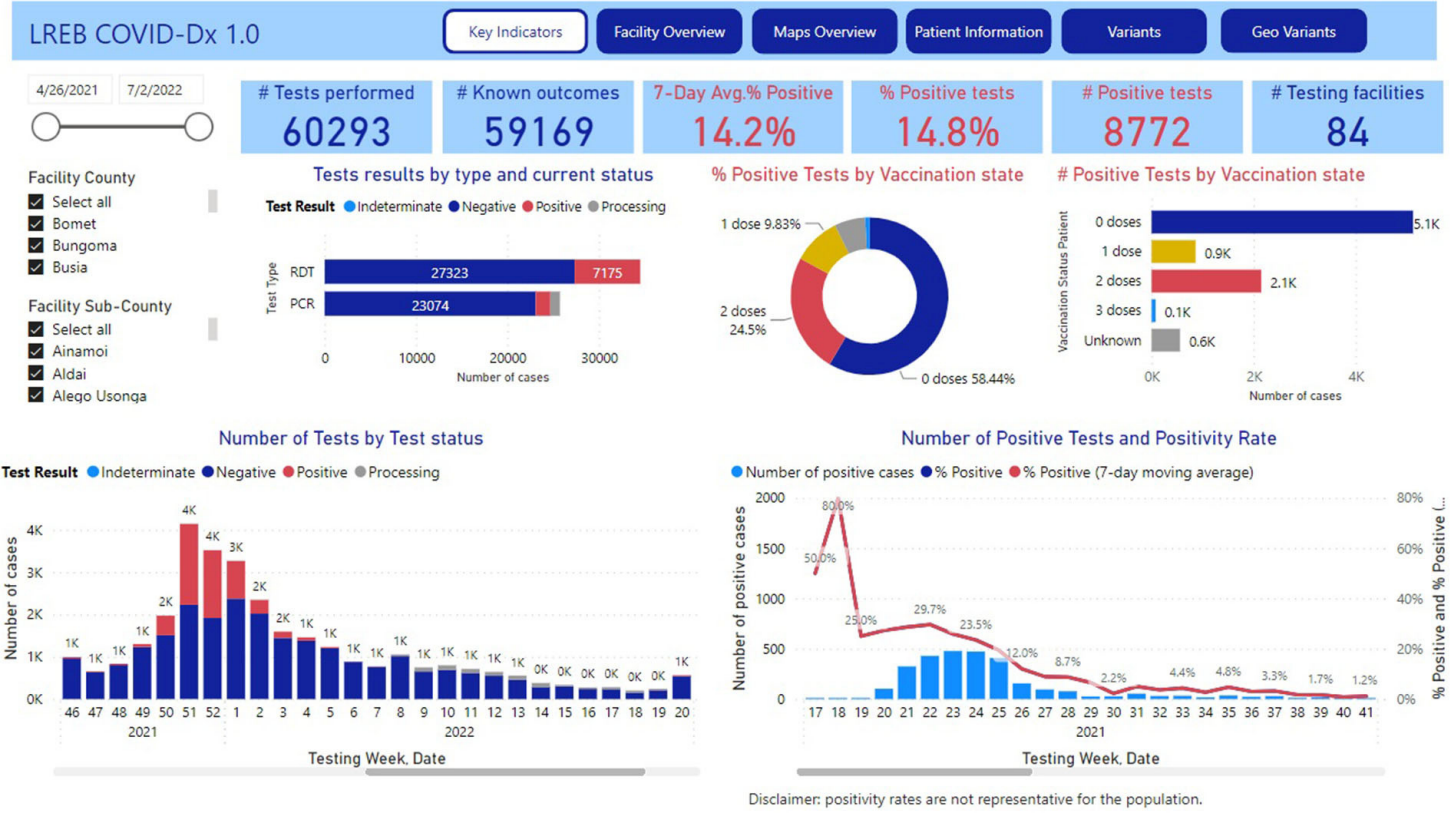
COVID-Dx dashboard main page.

COVID-Dx started with 9 private healthcare providers in Kisumu town, grew to 33 providers in Kisumu County and eventually to 84 providers in Lake Region Economic Bloc (LREB), as of today. Almost 60,000 SARS-CoV-2 test results have been uploaded into its dashboard. A total of over 8,600 positive test results were obtained, representing an overall 15% positivity rate (Figure 10). In addition to socio-demographic parameters, vaccination status was registered as soon as these became available (Q1 2021).

The implementation of *COVID-Dx* appeared very successful and is moving toward a more general digital epidemic preparedness and monitoring approach. Local adoption was secured because COVID-Dx was truly co-designed with local stakeholders and therefore most user-friendly and stimulating to fill in. The fact that GDPR proof COVID-Dx works semi-real time and uploads can be seen directly through the personal mobile phones and tablets of local policy makers and health managers, added to its popularity, both amongst public and private providers as well as health managers. For example, through local admin rights, the sub-County disease surveillance officers in Kisumu were better empowered to supervise their teams, push reminders, and approve data for Kenya Health Information System (KHIS). Importantly, the data in COVID-Dx are and remain property of the Department of Health (DoH) of participating counties.

**Figure 11:**
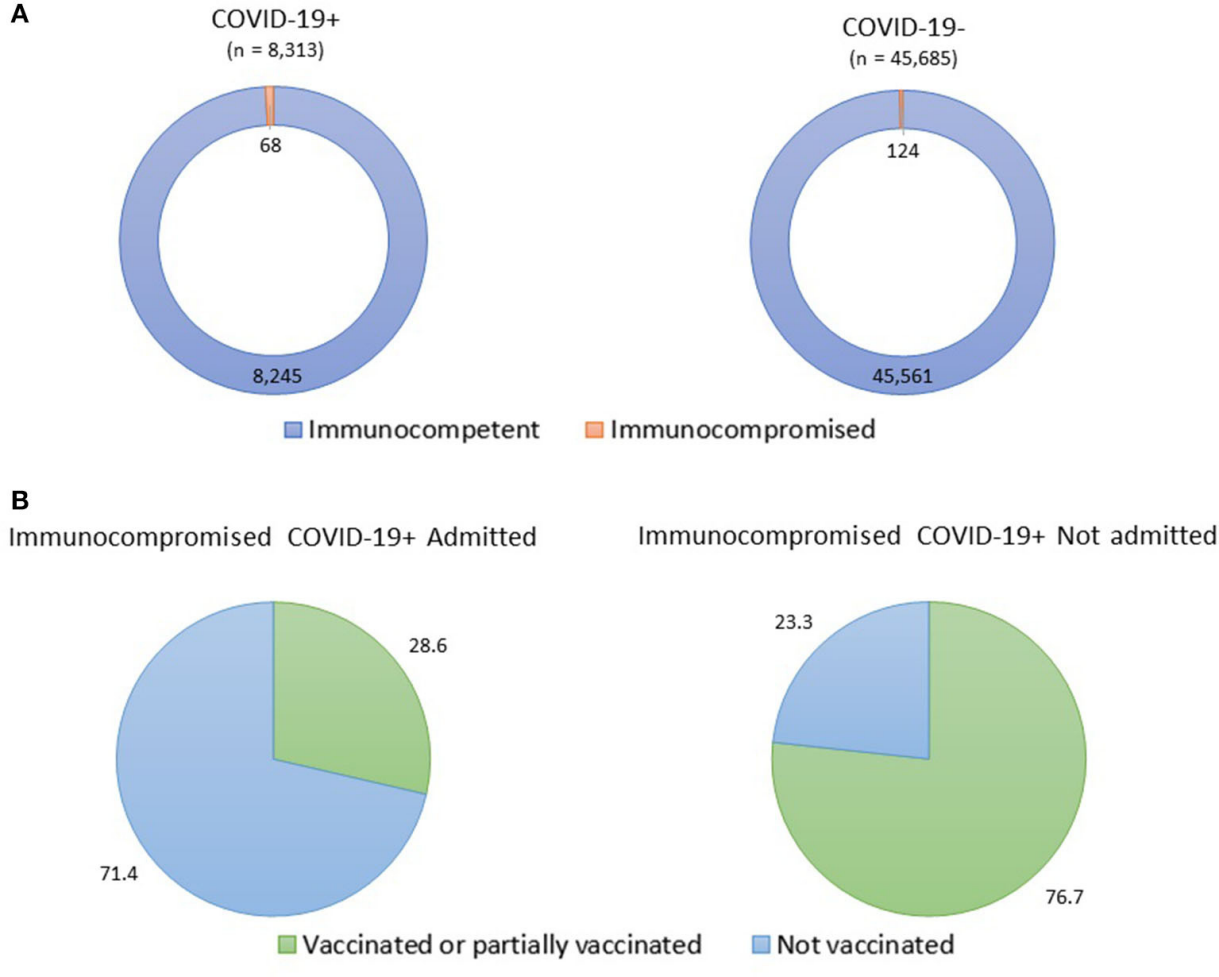
Prevalence of COVID-19 infections among immunocompromised patients. **(A)** Three times higher prevalence of immunocompromised status among COVID-19+ patients compared to non-COVID-19 patients; **(B)** Three times higher COVID-19 related hospital admission of unvaccinated immunocompromised patients.

The COVID-Dx dashboard is used extensively by policy makers and is resulting in regular policy briefs that are shared with pertinent stakeholders in LREB. For example, the very first COVID-19 Omicron patient in Kisumu was detected early December 2021, just 14 days after announcement of the Omicron variant by South Africa to the WHO, and immediately reported on the Department of Health website (61). End 2021 a disproportionate number of breakthrough infections were observed in health care workers. This led to a policy advice on December 28, 2021, for accelerated booster vaccination of this target population in LREB. The COVID-Dx dashboard indicated geographic areas in LREB of insufficient access to PCR (molecular)-based SARS-CoV-2 testing. This initiated a rapid field evaluation of alternative rapid diagnostic tests (RDT) for usage at the point-of-care (62), resulting in a first Kenyan publication in this field. Recent analysis on immunocompromised patients indicated that the proportion of such patients among COVID-19+ was significantly 3 times higher than among the COVID-19- individuals (*P* < 0.001). Also, it was found that the odds of being diagnosed COVID-19 were 2.3 higher in immunocompromised compared with immunocompetent patients (*P* < 0.001), see Figure 11A.

Moreover, among immunocompromised patients, the proportion of vaccinated (≥2 doses) or partially vaccinated (1 dose) individuals was higher (76.7%) in the COVID-19+ immunocompromised patients not admitted in the hospital, compared with the COVID-19+ immunocompromised individuals admitted in the hospital (28.6%) (*P* = 0.017), see Figure 11B. Also, we found that among immunocompromised COVID-19+ patients, the vaccinated or partially vaccinated individuals had 60% less likelihood of being admitted in the hospital than the not vaccinated individuals (*P* = 0.03). All these observations led to the recent LREB policy recommendation of including COVID-19 vaccination into the standard package of care for immunocompromised patients.

## Discussion

This paper provides a comprehensive overview of digital health interventions directed at overall health system strengthening in Africa to cope with the impact of the COVID-19 pandemic. It provides ample examples of how ongoing digital health systems strengthening efforts in Africa can be used to rapidly develop responses to emergency outbreaks, such as the COVID-19 epidemic. Apart from the general advantages of digitization of African health systems (real-timeliness, quality, transparency, efficiency) (41), this proven epidemic preparedness is an important additional asset. Based on our experiences with implementing multiple digital COVID-19 add-ons to ongoing health systems strengthening interventions, we can distill a number of lessons learned per WHO health strengthening building blocks and provide recommendations to further enhance the resilience of African health systems.

The *SafeCare4COVID app* (health work force—building block 1) showed how building on existing tools allows to rapidly reach out to health workers during the early stages of an epidemic, offering them greatly needed advice and support in adequately responding to a health crisis. Such tools may be especially useful during the onset of an epidemic. However, as experienced during later stages of COVID-19, the utilization of the app tapered off.

Digital platforms are particularly instrumental in immediate outreach to providers as well as patients and payers—as illustrated by the successful adaptation of the *MomCare program* (service delivery—building block 2), which managed to keep adherence to the continuum of maternal care at par during the peaks of the COVID-19 pandemic. Notably, where access to care was constrained, this mostly pertained to faltering supply chains of protective products, medical equipment and supplies, diagnostics, and drugs, as well as lack of information for patients and providers. Alleviation of such situations can be most rapidly achieved by digital health systems, which can expand and contract according to the real-time dynamics of an epidemic.

The lack of finance to private healthcare providers (finance—building block 3) is particularly worrisome during epidemics as the resources to fund working capital plummet when demand temporarily decreases in response to fear, dropping incomes and containment measures such as lockdowns. Digital financing schemes such as the mobile money-based *MCF Cash Advance program* offer effective means of providing rapid funds while avoiding the need to travel to a bank office during travel restrictions, as well as lengthy procedures for loan approval. As such, it provided a safety net for African healthcare private SME's to help bridge their COVID-19 inflicted financing gaps, while having less income and avoid foreclosure, keeping the healthcare supply-side afloat.

*CovidConnect*, aimed at increasing access to COVID-19-related diagnostics and medicines in Ghana, Kenya, and Nigeria (medical products, vaccines, and technology—building block 4), showed that smartphone-based Apps limit the reach of such efforts to a small part of the population in need. As a solution to this drawback, we performed a phone USSD SMS survey test instead. However, the latter had a very low response rate. Perhaps the timeframe during which this service was needed had already passed, or the stigma and fears of the consequence of a positive COVID-19 test led to its low response rate.

Epidemic preparedness requires action and preparations *before* an outbreak occurs, including setting up health information systems (building block 5) in anticipation of unknown emergencies. African policy-makers (Governance—building block 6) were at a great disadvantage during much of the early stages of the COVID-19 pandemic, because of an almost complete lack of data on prevalence, incidence, geographic location, target groups preferentially infected, among other very relevant information. As the *Diaries study* showed, pre-existing data collection efforts enable detailed insights right from the onset of a new health crisis, without the need for lengthy procedures to design a survey and a sampling frame, request ethical clearance and select, recruit and train interviewers. The COVID-Dx project, on the other hand showed how co-designed solutions and products and making the data property of the local health authorities, helped to scale up the local adoption of this tool. It also showed how semi-real time data, easily accessible in mobile devices in a secure format, are greatly valued by policy-makers.

## Actionable recommendations

Finally, our experiences during the COVID-19 epidemic have provided us with additional learnings pertaining to the financing and strengthening of health systems at large, yielding the following policy recommendations.

First, it proved challenging to secure rapid funding from institutional donors for quick adaptations and add-ons, due to their relatively intricate responsiveness to the emergency situation and subsequent prolonged lead times resulting from standard administrative processes. In that reality, it appeared more effective to move toward *ad-hoc* funding with dedicated private donors and foundations, particularly during the very first phase of emergency—even if those typically involve relatively small amounts, short timeframes, and each of those a different reporting format. The structural support of PharmAccess by the Netherlands Ministry of Foreign Affairs with flexible funding proved indispensable for quick action.

Second, in the very first phase of health emergencies, vertical (disease-specific) funding is needed and often available for immediate action. However, such funding should not become the norm and even during this first phase thinking should already be in the direction of “horizontalizing' funds.” For example, emergency COVID-19 funds could in the longer run be integrated into African health insurances, either directly or through an Epidemic Risk Equalization mechanism. Likewise, piloting new and rapid digital health system interventions in the emergency phase should preferably be performed with a scalable health systems model in mind; concurrent cost(-benefit) analyses and business modeling of such pilots is helpful.

Third, public-private partnerships are powerful scaling options in LMICs where private sector healthcare services are a substantial part of the health system. Although about half of African healthcare is provided through private healthcare providers, the private sector was initially (and for a long period) left out from government responses and policies. A purely public sector approach greatly reduces a country's potential to adequately deal with pandemics. Therefore, we recommend that early pandemic combat efforts should be geared toward crowding in the private sector response and make them complementary to public sector efforts, rather than replacing those (crowding out).

Fourth, efforts should be built on collaboration with local partners and stakeholders (providers, governments, donors, policy makers) to be optimized and avoid failure—our interventions that were most rapidly implemented and adjusted were those that could benefit from local ownership and support. Relatedly, the direct, one-on-one translation of tools that work in different cultural contexts does not always work effectively for the African reality (e.g., Luscii). Co-creation of tools with local LMIC stakeholders increases sustainability, ownership and success.

Fifth, new interventions and add-ons should be monitored closely, while teams on the grounds should be prepared to rapidly adjust whenever local circumstances require so. Digital systems can be particularly useful in this respect, since they provide almost real-time data for monitoring and follow-up. To reap the full benefits from digitalization, it is important to move beyond mere data collection and translate data into information that is timely, easily interpretable, relevant and useful for providers, policymakers and others—as shown e.g., by our dashboards.

Finally, demand and *suppl*y (as *well* as health and economics) go hand in hand. At the household level, strong containment measures kept the virus from spreading but came at the expense of households' income-generating capacity, leading many to fall into temporary poverty. Within the health sector, clinics needed technical support, equipment and PPEs to adequately preparing for COVID-19 patients, but without financial support and hampered by supply chain faltering, they could not properly invest in the required PPEs and keep their working capital at par.

## Conclusion

All in all, this paper provides important lessons for setting up emergency responses to a sudden outbreak of a pandemic (COVID-19) in LMIC settings, in particular Africa. The key message is that digital interventions aimed at general health systems support have the invaluable added value of flexibility that is needed to quickly react to sudden challenges, integrate specific epidemic responses as well as rapid assessment options for impact. Moreover, digital health system interventions proved to cross-pollinate different stakeholders of health systems with complementary benefits. Digitalization of health systems empowers their more virtual management and avoids time consuming and physically risky meetings in person during a contagious epidemic. Direct access through digital tools to health system players (providers, patients, households, payers, and policy makers) proved particularly important in times of travel restrictions and curfews, as was the case during subsequent five waves of COVID-19 in Kenya.

## Data availability statement

The raw data supporting the conclusions of this article will be made available by the authors, without undue reservation and following our data protection policies.

## Ethics statement

The studies involving human participants were reviewed and approved by the AMREF Ethical and Scientific Review Board. Research permits were obtained from the National Commission for Science, Technology and Innovation agency of Kenya (MomCare); and The Kenya Medical Research Institute: Scientific and Ethical Review Unit (SERU) KEMRI/SERU/CGHR/05/05/4038, and Jaramogi Oginga Odinga Teaching and Referral Hospital (JOOTRH) Ethical Review Board (ERB) IERC/2030/2020 (COVID-Dx). The patients/participants provided their written informed consent to participate in this study.

## Author contributions

NS, WJ, and TR conceived and wrote the first abstract and outline of the manuscript and conceived and contributed to several of the projects reported here. MD supported all PharmAccess COVID-19 response efforts and projects in Ghana, Kenya, Nigeria, and Tanzania reported here. MA lead and contributed to the projects in Ghana reported here. AG lead and contributed to the SafeCare4Covid project across Africa and other settings. GG-P contributed with data analytics of the SafeCare4Covid project and health diaries study, and contributed to the writing of the final draft of the manuscript. EM participated and contributed to several Kenyan projects reported here. NM, DG, RS, and DM lead and contributed to the Medical Credit Fund project. HM lead and contributed to several Tanzanian projects reported here. NN lead and contributed to several Nigerian projects reported here. WO lead and contributed to several Kenyan projects reported here. TD and EW participated and contributed to the MomCare project. MG, SK, and CK provided data management and analytic contributions to several of the projects reported here. KR provided insightful inputs during the execution of the Kenyan projects reported here. All authors have contributed, gave inputs to the writing, and graph design of their corresponding projects and have reviewed and approved the final version of the manuscript.

## Funding

The research reported here has received funding from the Ministry of Foreign Affairs of the Netherlands, Achmea Foundation, FDOV (Facility for Sustainable Entrepreneurship and Food Security), Joep Lange Institute, John Martin Foundation, MSD for Mothers, Postcode Loterij, Pfizer Foundation, and The ELMA Relief Foundation.

## Conflict of interest

Authors TR, MD, AG, MG, TD, SK, CK, GG-P, NS, MA, EM, HM, NN, WO, and EW were or are employed by PharmAccess Foundation. The remaining authors declare that the research was conducted in the absence of any commercial or financial relationships that could be construed as a potential conflict of interest.

## Publisher's note

All claims expressed in this article are solely those of the authors and do not necessarily represent those of their affiliated organizations, or those of the publisher, the editors and the reviewers. Any product that may be evaluated in this article, or claim that may be made by its manufacturer, is not guaranteed or endorsed by the publisher.

## Footnotes

1 Gómez-Pórez GP, de Graaff A, Dekker JT, Agyei BB, Dada I, Milimo E, et al. A digital tool approach to accelerate epidemic preparedness in limited-resource settings: Results from a multi-country study of COVID-19 readiness of healthcare facilities in Africa. (Submitted, 2022).
